# Refractory tracheoesophageal fistula treated using multi-stage surgery: A case report

**DOI:** 10.3389/fped.2022.1053154

**Published:** 2022-12-21

**Authors:** Yoichi Nakagawa, Satoshi Makita, Hiroo Uchida, Akinari Hinoki, Chiyoe Shirota, Wataru Sumida, Hizuru Amano, Masamune Okamoto, Aitaro Takimoto, Seiya Ogata, Shunya Takada, Daiki Kato, Yousuke Gohda, Yaohui Guo

**Affiliations:** ^1^ Department of Pediatric Surgery, Nagoya University Graduate School of Medicine, Nagoya, Japan; ^2^ Department of Rare/Intractable Cancer Analysis Research, Nagoya University Graduate School of Medicine, Nagoya, Japan

**Keywords:** tracheoesophageal fistula (TEF), esophageal atresia, thoracoscopy, recurrence, reconstruction

## Abstract

A tracheoesophageal fistula (TEF) recurs in approximately 2%–13% of cases of esophageal atresia with TEF that are treated surgically. Currently, there is no consensus on the most effective treatment to prevent recurrent TEF (RTEF). Herein, we present a patient with type C esophageal atresia who underwent thoracoscopic esophago-esophageal anastomosis and TEF repair at 2 days old. However, RTEFs were observed at ages 3, 6, and 11 months, and thoracoscopic TEF repairs using a pleural patch, fascia lata graft, and pectoralis major myocutaneous (PMMC) flap were performed, respectively. A fourth recurrence led to mediastinitis, shock liver, disseminated intravascular coagulopathy, and a compromised respiratory status. Hence, laparoscopic esophageal transection was first performed to improve the respiratory condition by preventing the regurgitation of gastric contents. Once the patient was stable, a subtotal esophageal resection with TEF closure followed by gastric tube reconstruction was performed. In conclusion, we encountered a case of refractory RTEF that was repaired four times using various techniques, including a fascia lata graft and PMMC flap. However, TEF still recurred after these four operations. The final surgical strategy involved an esophageal transection as a palliative therapy, which improved the respiratory condition, followed by closure of the TEF and subtotal esophageal resection. Finally, esophageal reconstruction using a gastric tube after the complete remission of inflammation was effective. This multi-stage surgery was considered the only choice to rescue the patient and effectively prevent another recurrence.

## Introduction

Tracheoesophageal fistula (TEF) recurs after approximately 2%–13% of surgeries performed to treat esophageal atresia (EA) with TEF (1–4). Postoperative recurrent TEF (RTEF) is categorized into three types (5): (i) congenital TEF, which is usually missed and unrepaired; (ii) RTEF, which reoccurs in the same location after repair; and (iii) acquired TEF, which forms a new pathway in a new location. The median onset time of RTEF is 2–5 months after the primary TEF repair (3, 6, 7). There are various reports regarding the treatment of RTEF; however, there is no consensus on which treatment prevents RTEF most effectively. We encountered a case involving refractory RTEF, which eventually resulted in mediastinitis with disseminated intravascular coagulopathy (DIC), shock liver, and respiratory failure. RTEF was repaired four times using various techniques, including a fascia lata graft and pectoralis major myocutaneous (PMMC) flap. However, TEF still recurred after these four operations, requiring subtotal esophageal resection with gastric tube reconstruction. Herein, we present this rare case of refractory RTEF and our treatment of this patient.

## Case description

**Figure 1:**
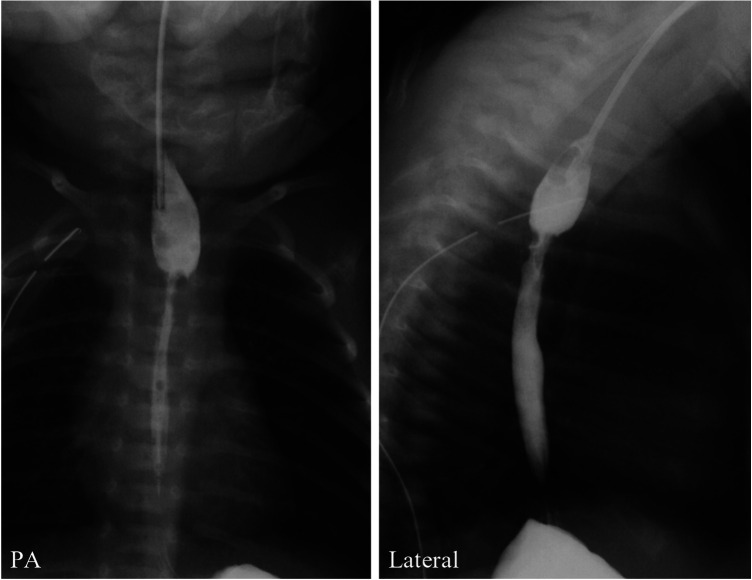
Postoperative contrast examination of the esophagus shows no apparent anastomotic leakage but anastomotic stenosis.

The patient was a female infant who was born at a gestational age of 36 weeks, weighing 1940 g, and diagnosed with type C EA. She underwent a thoracoscopic esophago-esophageal anastomosis and TEF closure with 4-0 absorbable braided suture at 2 days old. The postoperative contrast examination of the esophagus showed no apparent anastomotic leakage. However, asymptomatic anastomotic stenosis was observed (Figure 1), and the patient was discharged on postoperative day (POD) 31 without any complications.

**Figure 2:**
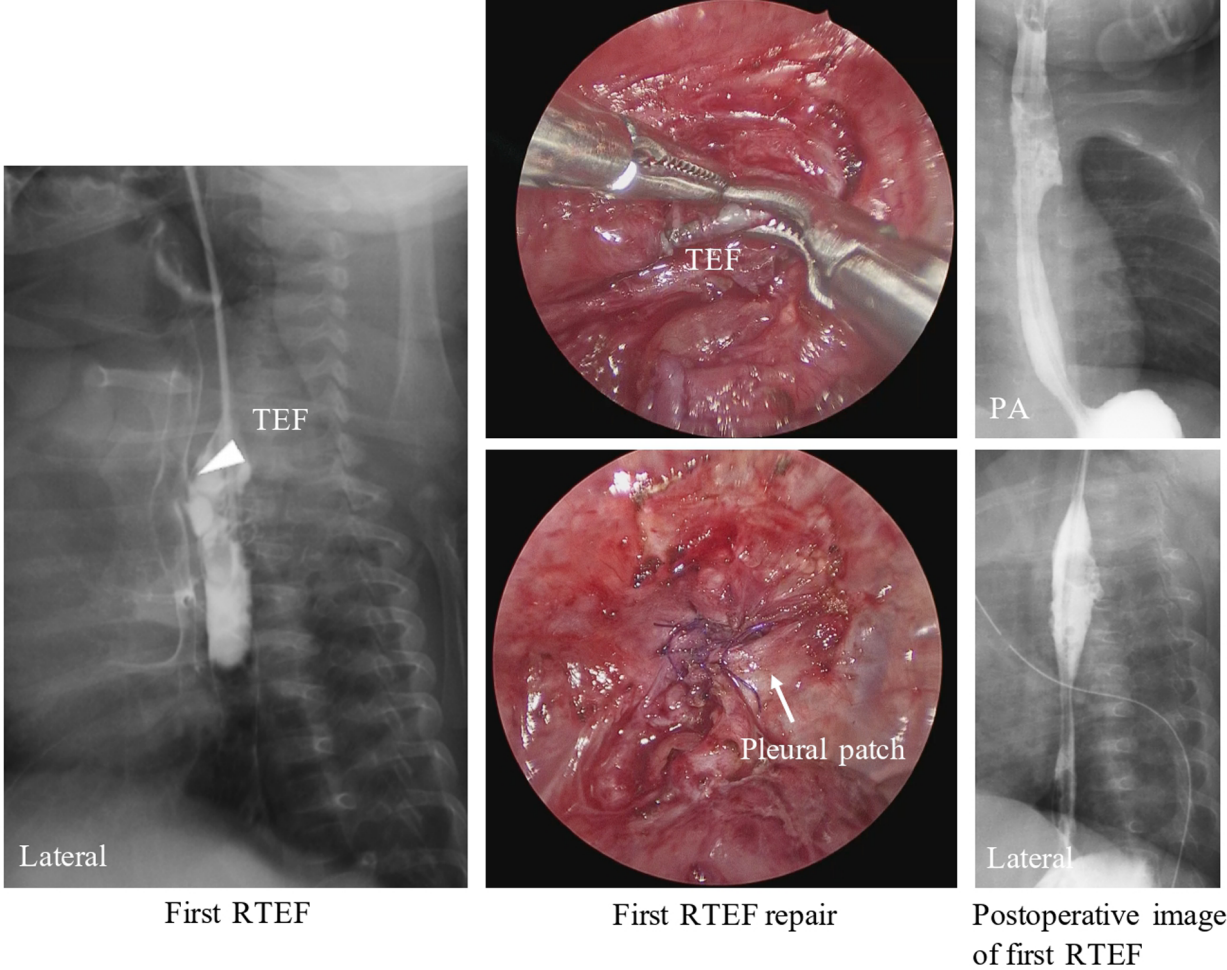
A contrast examination of the esophagus 2 months after the primary surgery shows an RTEF at the proximal site of anastomosis (arrowhead). The TEF was closed with sutures and the placement of a pleural patch (arrow) at the first RTEF repair. A contrast examination of the esophagus after the first RTEF surgery shows no apparent recurrent TEF. RTEF, recurrent tracheoesophageal fistula.

The patient was breastfed orally after discharge without any issues. However, at the age of 3 months, she presented with choking on breastfeeding and repeated upper respiratory infections. At the time, a contrast examination of the esophagus showed an RTEF (Figure 2: first RTEF). The patient underwent a thoracoscopic RTEF repair at 4 months old. The RTEF was closed with a 4-0 absorbable braided suture with the placement of a pleural patch (Figure 2: first RTEF repair) after confirming the esophageal suture line and that the trachea and esophagus were not attached. The anastomotic stenosis was not simultaneously treated since it was asymptomatic, and a contrast examination of the esophagus on POD 6 showed no apparent RTEF (Figure 2: postoperative image of the first RTEF). The patient was discharged on POD 12 without complications.

**Figure 3:**
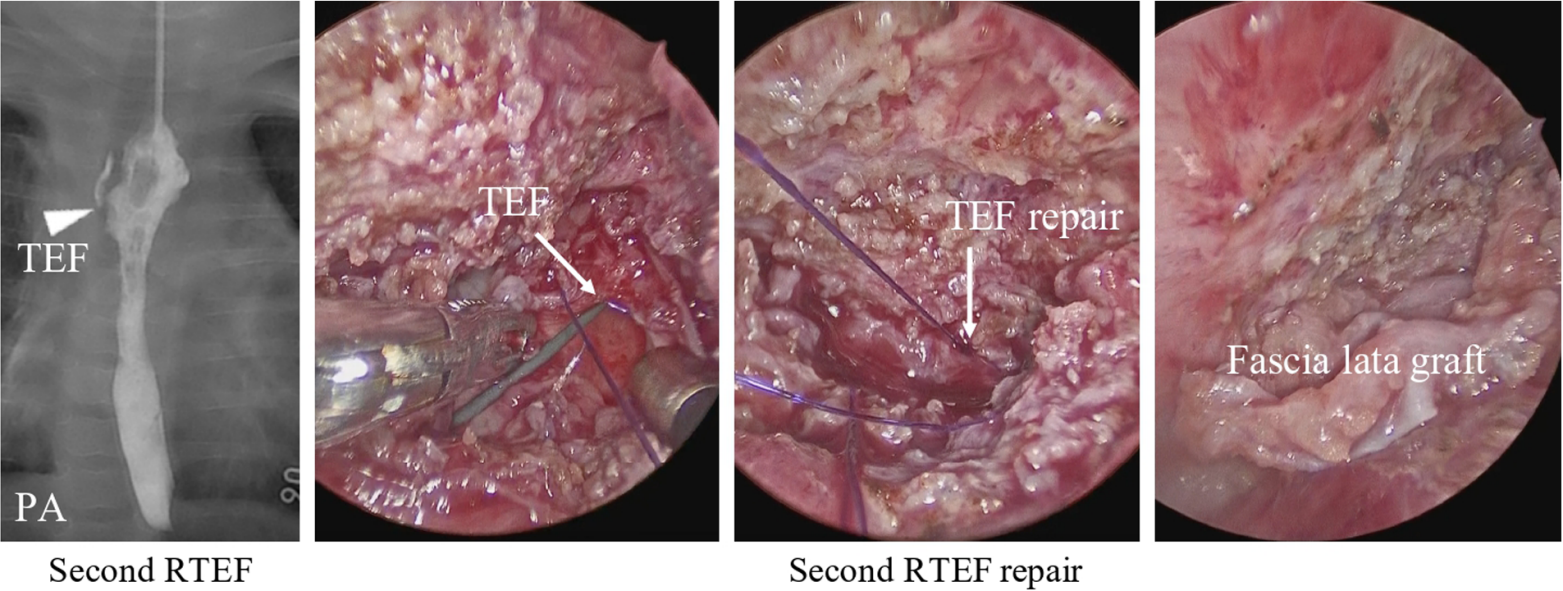
A contrast examination of the esophagus shows the second RTEF at the exact location of the first RTEF (arrowhead). During the second RTEF repair, the TEF was closed with sutures with a fascia lata graft (arrow). RTEF, recurrent tracheoesophageal fistula.

However, at 6 months old, the patient was reported to choke while breastfeeding. A contrast examination of the esophagus showed a second RTEF (Figure 3: second RTEF). Hence, the patient underwent a thoracoscopic RTEF repair with sutures and a fascia lata graft (Figure 3: second RTEF repair). The postoperative course was uneventful, and the patient was discharged on POD 19 without contrast examination.

**Figure 4:**
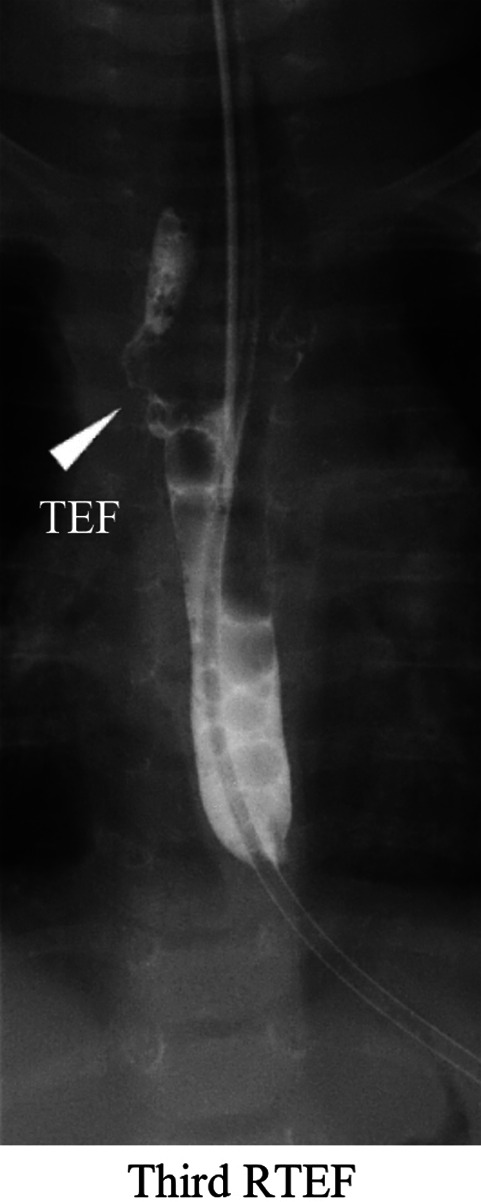
A contrast examination of the esophagus shows the third RTEF at the exact location of the first and second RTEFs (arrowhead). RTEF, recurrent tracheoesophageal fistula.

**Figure 5:**
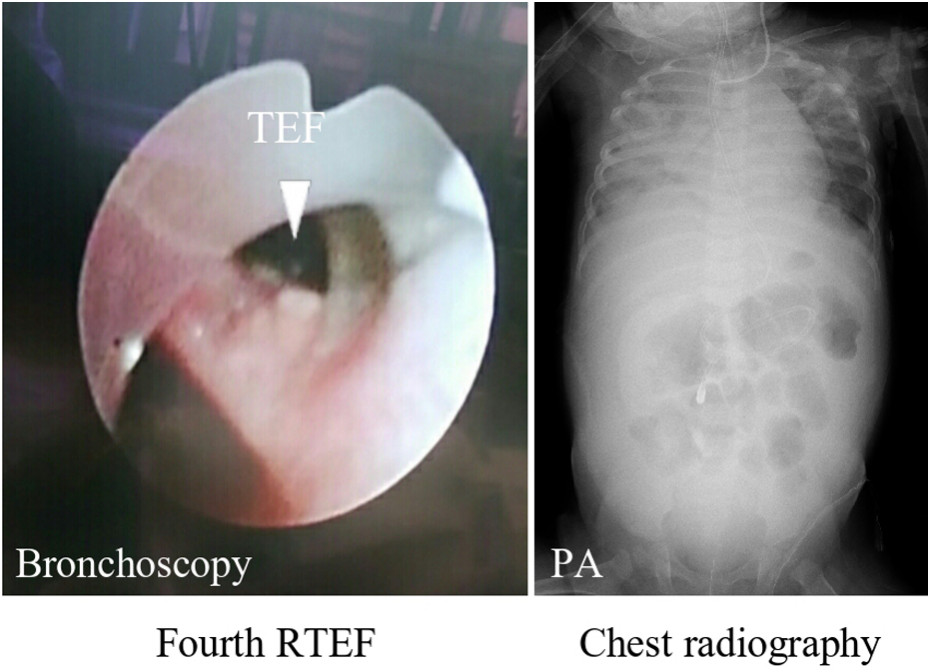
Bronchoscopy shows the fourth RTEF with a big hole (arrowhead). Chest radiography shows severe mediastinitis and right pneumonia. RTEF, recurrent tracheoesophageal fistula.

A contrast examination of the esophagus at 7 months old showed a third RTEF (Figure 4). Hence, oral intake was stopped completely, and the patient was tube-fed an elemental diet. After achieving adequate body weight gain at 11 months old, an RTEF repair was reattempted using a PMMC flap through a right thoracotomy. The patient was extubated on POD 22 but was reintubated due to a worsening of the respiratory condition. At the time, a bronchoscopy showed a fourth RTEF (Figure 5: fourth RTEF). The patient's condition gradually worsened, developing mediastinitis with DIC, shock liver, and respiratory failure (Figure 5: chest radiography). The laboratory parameters were abnormally high, with aspartate aminotransferase levels of 25,625 U/L, alanine transaminase levels of 5,874 U/L, and procalcitonin levels of 58.0 ng/ml. Gastric reflux and RTEF were considered to be the main causes of mediastinitis with respiratory failure. However, the patient was not stable enough to undergo radical surgery. Therefore, the patient underwent a laparoscopic esophageal transection with gastrostomy as a palliative therapy on POD 32 to improve her respiratory condition. The respiratory condition gradually improved after the esophageal transection. Subsequently, an esophageal subtotal resection and RTEF repair through a thoracotomy were performed on POD 37. The RTEF was closed with sutures and wrapped with another PMMC flap. The patient was extubated on POD 52 and discharged on POD 98 (aged 1 year 3 months).

At 1 year 7 months old, the patient underwent a laparoscopy-assisted substernal gastric tube reconstruction. The gastric tube was made using a linear stapler with a 3-cm diameter. Again, no recurrent laryngeal nerve palsy was reported. Now, at the 1.5-year follow-up, the patient can eat food orally.

## Discussion

Of particular importance in treating RTEF is no recurrence of RTEF; however, in this case, RTEF occurred four times in total, finally requiring a subtotal esophageal resection with substernal gastric tube reconstruction. No apparent cause of RTEF was observed when reviewing the operation video and perioperative management. Although anastomotic leakage has been reported to be the main cause of RTEF (5, 8, 9), in this case, esophago-esophageal anastomosis was considered to have caused no apparent problem by postoperative course. A previous study has reported that RTEF can occur from inflammation due to leakage in the trachea when a TEF is single-ligated using a 4-0 absorbable braided suture (3). Hence, the ligation of TEF in this patient was changed from single- to double-ligation. The first and second RTEFs were closed with sutures and wrapped with a pleural patch and fascia lata graft, respectively; however, these were ineffective. We subsequently reflected that a strong and more vascularized flap should have been used instead of a pleural patch or fascia lata graft. RTEF is commonly repaired by interposing a vascularized flap between the TEF repair site and the esophagus. Such vascularized flaps include the pericardium, pleura (5, 7), omentum (10), and muscles, such as intercostal (11), latissimus dorsi (12), and sternothyroid (13) muscles. The pleural patch (5, 7) and fascia lata graft (14) are useful for RTEF; however, in hindsight, a vascularized flap could have been more effective for the second RTEF in this case. A total of 56 patients underwent thoracoscopic TEF repair at primary surgery between August 2013 and September 2022 at our institution; however, RTEF occurred only in this patient. Furthermore, thoracoscopic esophageal atresia surgery at our institution is performed by an experienced surgeon who had performed successful thoracoscopic RTEF repairs at another institution. Initially, a thoracoscopic TEF and RTEF repair was preferred at our institution; however, a thoracotomic RTEF repair is now considered necessary as early as possible after experiencing severe complications, as in this case.

The thoracotomic RTEF repair was performed at the third RTEF, and repairing the third RTEF with a PMMC flap was successful. The patient's respiratory condition gradually improved postoperatively, leading to extubation. However, a fourth RTEF quickly recurred after the third RTEF repair. Thus, we should have considered that the patient needed to undergo esophageal resection with interposition to repair and prevent the fourth RTEF. This was because RTEF had recurred three times despite employing various techniques; then, the patient would not have been in critical condition. Hence, a laparoscopic esophageal transection was selected as a palliative therapy to prevent gastroesophageal reflux. This procedure was selected because severe adhesions around the esophagus were predicted, and the patient could not withstand a lengthy operation. Once the respiratory condition improved and the patient was stable, a subtotal esophageal resection with RTEF closure was performed, and the RTEF was wrapped with a new PMMC flap. This multi-stage surgery was considered the only choice to rescue the patient and effectively prevent another recurrence. Finally, esophageal reconstruction with a gastric tube was performed, after complete remission of mediastinal inflammation, *via* a substernal route to avoid severe adhesions in the mediastinum and the risk of a fifth RTEF.

## Conclusion

We encountered a case of refractory RTEF involving various surgical techniques performed to repair the RTEF; however, RTEF occurred four times in total. Severe inflammation was considered the main mechanism of RTEF. The final surgical strategy, in this case, involved an esophageal transection as a palliative therapy to prevent the regurgitation of gastric contents and improve the patient's respiratory condition, followed by closure of the TEF and subtotal esophageal resection. Finally, esophageal reconstruction using a gastric tube after the complete remission of inflammation was effective.

## Data Availability

The original contributions presented in the study are included in the article/Supplementary Material, further inquiries can be directed to the corresponding author.
